# Carbon-Based Nanomaterials as Promising Material for Wastewater Treatment Processes

**DOI:** 10.3390/ijerph17165862

**Published:** 2020-08-13

**Authors:** Krzysztof Piaskowski, Paweł K. Zarzycki

**Affiliations:** Faculty of Civil Engineering, Environmental and Geodetic Sciences, Koszalin University of Technology, Sniadeckich 2, 75-453 Koszalin, Poland; Krzysztof.piaskowski@tu.koszalin.pl

**Keywords:** graphene oxide, activated sludge, wastewater treatment, Raman spectrophotometry, principal components analysis

## Abstract

In the latest literature search, the technology based on graphite oxide (GO) nanomaterials exhibits a great potential in many aspects of wastewater treatment involving adsorption, photocatalysis, disinfection and membrane process. In this study experimental data involving the carbon element in different forms such as active carbon (AC), graphite and graphene oxide (GO) applied as the active reagents in wastewater treatment are summarized and discussed. The first step was to characterize the aforementioned carbon materials and nanoparticles using various complementary techniques. These include optical microscopy, scanning electron microscopy (SEM), energy dispersive spectroscopy (EDS), Raman spectrophotometry and zeta potential measurements. The second issue was to design the relatively simple experiment enabling us to observe the physicochemical and biological effects of carbon nanoparticles in the presence of sewage water and/or active sludge. Obtained experimental data have been inspected using univariate and multivariate (principal component analysis, PCA) approaches confirming the complex interaction of GO nanoparticles with microorganisms that are present in activated sludge. This experiment enabled the collection of an initial data set to design different large scale investigations focusing on active nanoparticles affecting wastewater purification. PCA calculations clearly revealed that GO strongly affects the wastewater technological processes investigated. It is hoped that the described results will allow the design of smart environmental protection systems in the future.

## 1. Introduction

**Figure 1:**
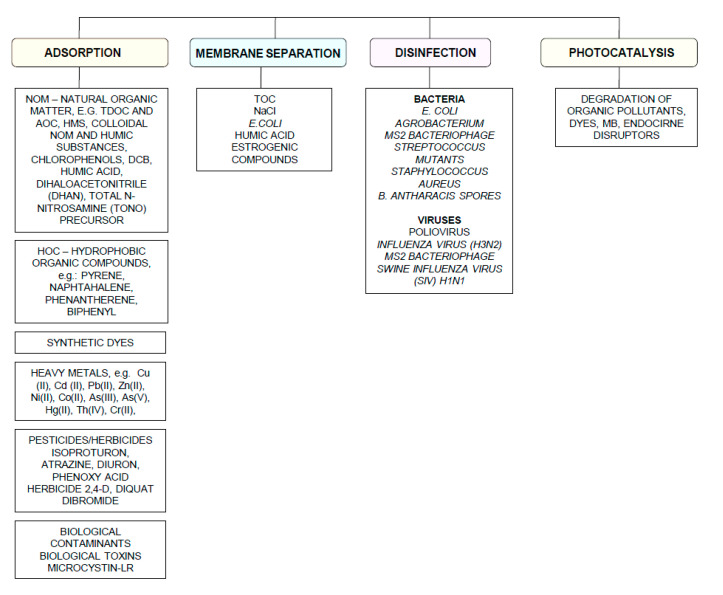
The potential applications of carbonaceous nanomaterials in wastewater treatment.

Carbon nanoparticles are presently considered as the most versatile materials that can be used for improvement of wastewater treatment processes. Extensive research carried out worldwide has resulted in the discovery of new carbon related materials that have been successfully implemented in wastewater treatment and environmental protection technologies [1,2]. According to the latest literature search, the main targets such as micropollutants or pathogenic microorganisms, which can be removed from wastewater by carbon nanoparticles in various technological processes, are summarized in Figure 1 [3,4,5]. The unique physical and chemical properties of carbon particles determine a wide range of practical applications [3]. The most commonly investigated carbon-based nanomaterials are graphene (G); graphene oxide (GO); single-walled carbon nanotubes (SWNT); and multi-walled carbon nanotubes (MWNT). These materials can be applied in their pure forms, or they may act as complex hybrid materials [4,5].

The next generation wastewater treatment and water supply systems relying on carbon nanomaterials and nanotechnologies should be environmentally friendly and economically feasible [6]. High surface area to volume ratio of nanomaterials enhances reactivity with environmental pollutants and pathogens like bacteria, fungi, or viruses [7]. An important branch of carbon based material is graphene derivative, namely graphene oxide (GO). This particle consists of an oxygen-containing functional group attached to the graphene layer. Such a structure, especially a high density of oxygen functional groups (e.g., carboxyl, hydroxyl, carbonyl, and epoxy) in the carbon lattice, enables GO to form stable suspensions in aqueous solution [8]. This property enables practical applications that are specific to processes occurring in the water environment. Moreover, GO is characterized by additional unique features including large specific surface area, selective and abundant adsorption sites, short intraparticle diffusion distance, tunable pore size, super charge carriers’ mobility, and outstanding electrical conductivity. Its mechanical strength and high adsorption capacity allows GO to form hybrid nanocomposites with various materials. Importantly this nanomaterial is characterized by simple manufacturing and low production cost [9,10].

Our previous research involving carbon materials was related to the adsorption properties of graphite and activated carbon particles focusing on sewage and water treatment technological processes [11]. Particularly, elimination of low-molecular mass synthetic dyes including malachite green, ponceau 4R and brilliant blue FCF were investigated [12]. Most recently, our group have synthesized graphene oxide nanoparticles using a modified Hummer’s method [13]. From raw GO water suspension, several solid forms were derived including air dried film (GO_AD) and lyophilized sponge (GO_L). The presented research is focused on physicochemical characterization of GO materials and the effect of such nanoparticles on wastewater treatment processes. This was performed using a batch test (jar test) in the laboratory based on biological treatment with activated sludge. The obtained data set was explored using a univariate approach and also discussed taking into account results of multivariate computation (principal components analysis).

## 2. Materials and Methods 

### 2.1. Chemicals

**Figure 2:**
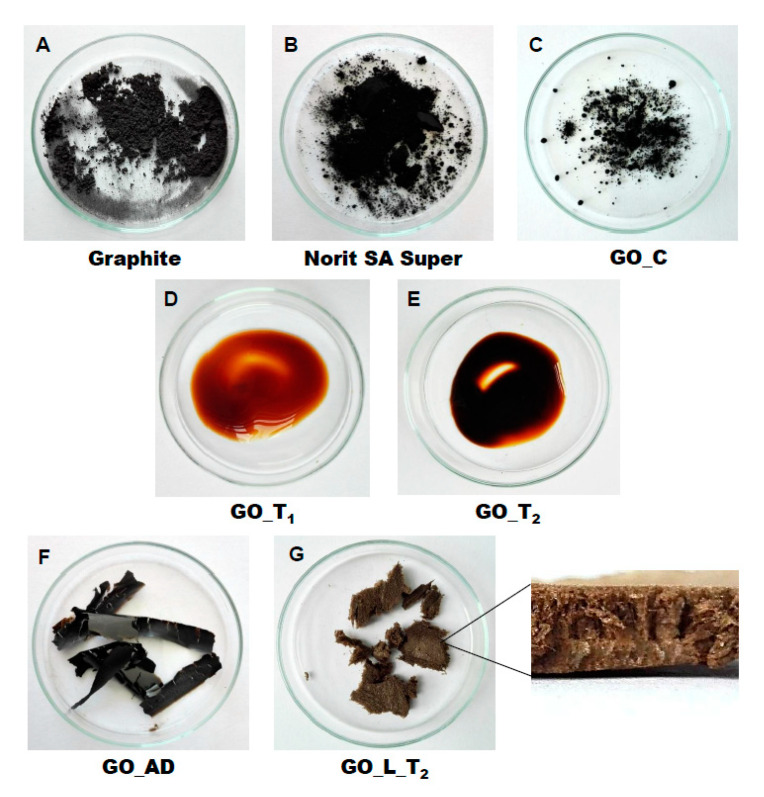
General view of investigated carbon materials: graphite (**A**), activated carbon Norit SA Super (**B**), commercial graphene oxide—GO_C (**C**), graphene oxide—GO_T_1_ (**D**), graphene oxide—GO_T_2_ (**E**), graphene oxide after air drying—GO_AD (**F**) and GO after lyophilization—GO_L (**G**).

Carbon materials used as raw chemicals, graphite (powder < 20 µm) and activated carbon (Norit SA Super) were obtained from Sigma-Aldrich (USA) and Cabot Concern, and distributed by Brenntag (Kędzierzyn Koźle, Poland), respectively. Graphene oxide standard (powder, 15–20 sheets, 4–10% edge-oxidized) was obtained from Sigma-Aldrich, USA. Moreover, graphene oxide (GO) was synthesized in our laboratory using a modified Hummer’s method according to the detailed protocol reported in [13]. From GO water dispersion we prepared a lyophilized GO form (sample freezing temperature −100 °C/1 h, drying time 18 h at temperature 25–29 °C and pressure 1–2 Tr) and air dried material (60 °C; 18 h). Additionally, our experiments involved water dispersed GO after 3 and 36 weeks from synthesis time (T_0_), labeled as GO-T_1_ and GO-T_2_ (Figure 2). All GO forms were stored at room temperature within sealed jars filled with air.

### 2.2. Batch Experiment

This process was based on a mixture of raw wastewater and excess sludge supernatant collected from “Jamno” Wastewater Treatment Plant (“J”WTP; located within Koszalin city area, Poland). Moreover, we used activated sludge biomass obtained from the biological aeration tank of “J”WTP. The batch experiment was performed within conical flask containers (250 mL) placed on the shaker (24 h, 150 rpm; Laboshake Gerhardt Typ RO 500, Germany) at room temperature. Using this experimental setup several mixtures were investigated including:

(1) 100 mL mixture of raw wastewater and sludge supernatant (1:1; *v*/*v*) combined with given carbon material at dose 500 mg/L. After reaction time (24 h) the resulting liquid was filtrated using cellulose filter (diameter 125 mm, medium type, producer Chemland, Poland) and analyzed.

(2) 75 mL mixture of raw wastewater and sludge water (1:1; *v*/*v*) combined with 75 mL of activated sludge (concentration 3.8 g/L) sample and given carbon material at dose 500 mg/L. After reaction time (24 h) resulting suspension was spitted into two parts. One volume (70 mL, approximately) was filtrated as above and the second volume (80 mL, approximately) was used for analysis of activated sludge parameters.

### 2.3. Sample Analysis

The morphology of the carbon materials was examined by SEM. The sample was mounted directly on the holders and then observed at 20 kV in a JEOL JSM-5500LV electron microscope (JEOL Ltd., Japan). Moreover, samples were investigated using an EDS system (Oxford Instruments, United Kingdom) that was a component of the JSM-5500LV electron microscope. Additionally, Raman spectroscopy was performed by Raman microscope DXR (produced by Olympus, software version: OMNIC Specta 2.2.43) operating under laser (4 W) analytical wavelength 455 nm. Water suspension samples were not dried before Raman analysis. 

Chemical analysis included pH, conductivity, total organic carbon TOC, total nitrogen TN, ammonium nitrogen, and orthophosphates. The analysis was made after sample filtration through hard paper filter. The pH and conductivity were measured by a CyberScan PCD6500 (Eutech Instrument, Singapore). The TOC and TN were measured by TOC Analyzer TOC-VCPH with TNM-1 unit from Shimadzu. The NH_4_-N determination was conducted using a direct Nesslerization method and the orthophosphates by colorimetric method with ammonium molybdate measured by UV-VIS DR 5000 spectrophotometer (HACH Lange, Germany). Zeta potential of graphene oxide samples in liquid form was measured using Zeta Pals (Zeta Potential Analyzer, Brookhaven Instruments Corporation, USA)

Activated sludge analysis included: (i) microscopic observation, (ii) biomass concentration (MLSS) and (iii) the volume of settled sludge after 30 min sedimentation (SSV_30_). Then, (iv) the settling properties using the sludge volumetric index after 30 min of sedimentation (SVI30) and (v) biological activity using dissolved oxygen uptake rate (DOUR) test were registered for measurements of the respiration rate of organisms within the biomass investigated. Finally, (vi) a capillary suction time (CST) test was used to measure the filterability and the ease of removing water from sludge (CST-meter Type CST-M02, from Envolab, Poland).

Quantitative data concerning investigated samples were inspected with PCA procedure using XLSTAT XLSTAT-Pro/3DPlot statistical and visualization package (version 2008.2.01) provided by Addinsoft (Paris, France) and working with Microsoft Excel 2002. The appropriateness of multivariate calculations for our data was assessed by performing the Bartlett’s sphericity test.

## 3. Results and Discussion

In general, classical carbon-based materials such as various activated carbons are still the most commonly used adsorbents in water and waste treatment technology for removing organic and inorganic pollutants [14] due to their high efficiency and simplicity of the technology behind. Disadvantages of these materials include relatively high production cost, low degree of regeneration and poor selectivity. Due to the well-known problem of low molecular mass organic micropollutants that are present in sewage like EDCs (endocrine disrupter compounds), there has been extensive research focusing on the invention of more efficient, selective and recyclable carbon sorbents [15,16].

These are predominantly designed to replace classical active carbons. Graphene oxide can be one of the nanoparticles of interest, taking into account the variety of oxygen-containing functional groups (hydroxyl, epoxy, and carboxyl) that may be attached to the GO layers. This can strongly affect adsorption properties of micropollutants and, particularly, GO selectivity to given low-molecular mass compounds’ adsorption during wastewater treatment [17,18]. Recently, our group have produced graphene oxide in different forms including water dispersion, dense air dried material or porous/spongy lyophilizate using a modified Hummer’s method (Figure 2). Detailed synthesis and drying protocols were reported in [13]. So far, we have found that drying protocol may strongly affect the physicochemical properties of the GO raw matrix.

### 3.1. Physicochemical Characterization of Carbon Materials

**Figure 3:**
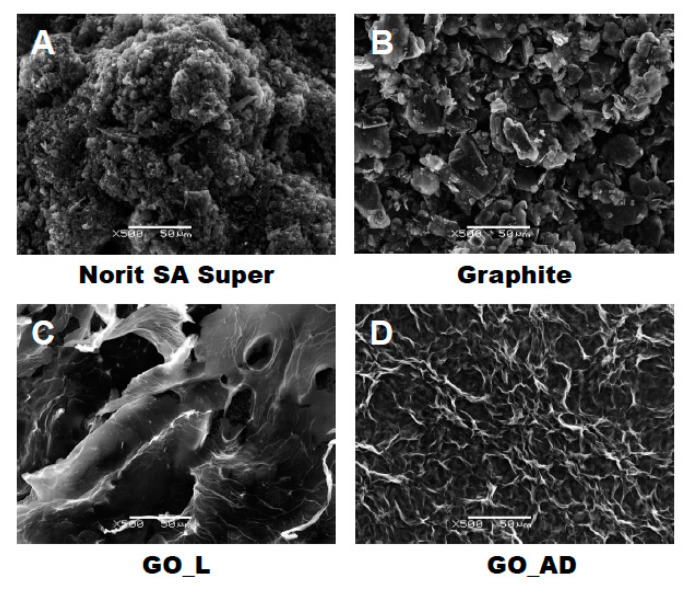
Scanning electron microscope (SEM) showing carbon materials used in laboratory tests: activated carbon Norit SA Super (**A**), graphite (**B**), graphene oxide after lyophilization—GO_L (**C**), graphene oxide after air drying—GO_AD (**D**).

As presented in Figure 2, investigated GO materials were obtained in different solid forms. Recently, we described dye separation results using an electrophoresis experiment in which the GO matrix may affect target analytes’ electromigration in different way, depending on the initial GO form tested [13]. Before conducting the present experiments related to nanoparticles’ effect on sewage treatment, we were interested in a more detailed analysis of the GO materials prepared. Figure 3 reveals large scale morphology of carbon particles (Norit SA Super, A; graphite, B) and graphene oxide materials (lyophilized, C; air dried, D) using the SEM technique. Norit SA Super (A) is strongly porous and graphite (B) consists of characteristic small carbon flakes. Both GO materials are composed of two-dimensional sheet-like and multiple lamellar layer structures, which seem to be different, similar to the macroscopic views presented in Figure 2F,G. These pictures were recorded by a visible light camera and indicate that the lyophilized form is more porous/spongy compared to the form of the thin films of GO after air drying. However, it should be mentioned that for higher SEM magnification the structures of both GO forms (lyophilized and air dried) are almost equal. It is noteworthy that the lyophilized form can be spontaneously dispersed in water, as previously mentioned [13]. Lyophilized GO can be reconstituted in water as a stable suspension, confirmed by the preliminary suspension experiment with both tap and distilled water at room temperature. Measured values of zeta potential were at a level of –38 mV for dispersed GO particles. This measurement confirmed the existence of a stable graphene oxide suspension due to the presence of a number of ligands such as ionized carboxylic groups on the GO surface. The consequence of such a chemical structure are electrostatic repulsive forces between individual particles, which allow formation of a stable suspension [19]. In comparison, under similar conditions (solvent, temperature, particle size) activated carbon cannot form a stable suspension [13]. We may speculate that easy GO dispersion can be an advantage for application of such material in the wastewater processes investigated.

To measure the surface composition in a more quantitative way, an energy dispersive spectroscopy (EDS) investigation was performed. Detailed elemental analysis provided by EDS is listed in Table 1. These data clearly indicate that graphite, which was the starting material for GO synthesis, consists of pure carbon. Norit SA Super is composed of carbon at high concentration (more than 94 at.%) and several elements, mainly present as impurities remaining after the activation process or intentionally added to obtain the given adsorption properties [12]. GO consists of a carbon matrix (more than 55 at.%) and oxygen as well as a low level of sulfur This is in agreement with results of GO materials reported in the literature by other authors, which were synthesized by different modified Hummers protocols. For example, Yoon et al. [20] have reported GO flakes consisted of more than 63% at. of carbon, 36 % at. of oxygen and 0.5% at. sulfur, whilst Al-Gaashani et al. [21] produced GO particles characterized by 58.56, 40.81 and 0.63 % at, respectively. From a practical point of view the above reported measurements confirm an ability of GO particles to attract water molecules due to the presence of oxygen containing ligands. Trace level sulfur element is recorded because of the synthesis protocol, in which sulfuric acid is commonly used. 

**Figure 4:**
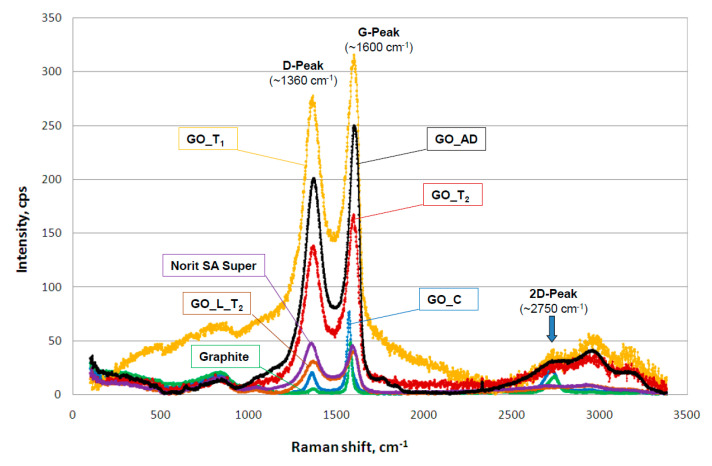
Raman spectra of carbon materials used in laboratory tests. Activated carbon Norit SA Super; commercial graphene oxide—GO_C, graphene oxide—GO_T_1_ (after 3 weeks from synthesis time); graphene oxide—GO_T_2_ (after 36 weeks from synthesis time); graphene oxide after air drying—GO_AD and GO after lyophilization—GO_L.

It has been found that our GO materials both in liquid and solid form may change color from light to dark brown during sample storage at room temperature over a few months. To investigate this phenomenon the Raman spectra were recorded for raw and stored sample batches. For this comparison we also measured the remaining carbon materials used in this study as well as the GO standard that was available commercially. Generally, Raman spectroscopy is widely used to characterize crystal structure, disorder, and defects in graphene-based materials. Results of analysis are presented in Figure 4, in which all Raman spectra from individual samples were combined. As can be seen, all materials exhibit characteristic peaks for layered carbon structures.

The specific Raman bands and their intensity ratios may provide useful information about the structure of carbon materials, particularly graphene particles. It has been reported before that the D-peak intensity can be used as a measure of the degree of disorder and the D/G band intensity ratio represents geometry defects including edges, vacancies, ripples, etc. [22]. The peak (at 2750 cm^–1^) denoted by label 2D-band is attributed to the development of graphene structure, while 2D/G may represent the number of layers in graphene. It has been documented that, if the 2D/G intensity ratio is less than 1, this may suggest a multi-layered graphene structure [23,24]. It should be mentioned that the oxidation process in GO synthesis produces structural defects which shift the physical properties of GO away from that of pure graphene [25].

Table 2 consists of calculated values of intensity ratio of D/G (I_D/G_) and 2D/G (I_2D/G_) bands derived from individual spectra in Figure 4. The lowest I_D/G_ value was recorded for the graphite sample, proof of the highly ordered structure of this carbon material. Commercially available graphene oxide (labeled as GO_C) is characterized by I_D/G_ value at a level of 0.26 whilst GO samples synthesized in our laboratory displayed much higher values close to 0.8. This may indicate the presence of carbon atoms with different hybridization and/or partial exfoliation resulting in increase of disorder. Decrease of I_D/G_ value during storage time strongly suggests reorganization of GO layers. This can also be confirmed by decreasing the overall band intensity visible for long-time stored GO samples in liquid form. As can be seen from data presented in Table 2 all reported values of I_2D/G_ parameter are below 1. In case of GO samples this may be interpreted as the presence of a multilayer structure of carbon particles. Moreover, considering similarity between the spectra shape of 2D peaks it can be concluded that for GO samples produced by our team the number of layers is similar for liquid and air dried forms. Interestingly, the lyophilization process may strongly affect GO structure. 

### 3.2. Jar Test Involving Carbon Materials, Wastewater and/or Activated Sludge 

Based on physicochemical investigations reported above we hypothesized that our GO samples may show different chemical and biological activity. We tried to verify this hypothesis performing simple jar tests involving real wastewater and activated sludge and then data mining using univariate and multivariate approaches. In general, due to the various functional groups located on the graphene oxide surface, GO has already been proposed as a potential adsorbent for metal ion complexation through both electrostatic and coordinate approaches. It has been reported that strong metal ions’ adsorption on the GO surface, in comparison to the adsorption effectiveness of pristine graphene, results from the high content of oxygen containing ligands [3,26]. It has been found that GO may exhibit one of the strongest adsorption properties for many water pollutants [27].

#### 3.2.1. Experiment Based on Carbon Materials and Wastewater

Data in Table 3 consist of the measured values of physicochemical parameters recorded using a jar test. This experimental setup consists of raw wastewater and all carbon materials were investigated with a fixed dose equal to 0.5 g/L. This particular level of carbon materials was selected considering our preliminary experiments indicated that significant effects of additives on physicochemical parameters can be detected under such jar test conditions. As can be seen, GO additive in liquid form and for both storage times (GO_T_1_ and GO_T_2_) significantly affects the values of the physicochemical parameters investigated, except concentration of orthophosphates, which are negatively charged. Decreased level of pH, conductivity, TOC, and IC as well as total nitrogen and ammonia nitrogen was observed for GO_T_1_ and GO_T_2_. This can be associated with the strong negative values of electrokinetic potential (zeta) of these nanoparticles, which was measured at a level of −33 mV. A similar effect (visible especially for TOC and positive charged ammonia nitrogen) is observed for negatively charged activated carbon particles (Norit Super SA; −20 mV) [13]. Interestingly, in spite of the high value of zeta potential measured for lyophilized graphene oxide (GO_L; −38 mV) [13], this material does not affect TOC, orthophosphates, total nitrogen and ammonia nitrogen (only pH, conductivity and IC). This can be explained by the worse dispersion of GO_L particles in reaction liquid in comparison to GO_T_1_ and GO_T_2_. TOC values were decreased for both GO_T_1_ and GO_T_2_ as well as Norit Super SA materials, whilst all GO additives resulted in decrease of inorganic carbon (IC) values.

#### 3.2.2. Experiment Based on Carbon Materials and Wastewater with Activated Sludge

The second jar test was intended to record carbon nanoparticles’ effect on biological wastewater treatment involving activated sludge. In this experiment two aspects were investigated simultaneously: (*i*) changes of the physicochemical parameters of treated wastewater and (*ii*) activated sludge parameters after reaction with carbon materials. In this case the final quality of treated wastewater should be the result of at least two synergistic effects, namely the physicochemical impact (adsorption or oxidation) of carbon materials on pollutants present in the wastewater, and the microbiological decomposition and/or transformation of pollutants through activated sludge modified by carbon material additives. It is noteworthy that the main factor enabling wastewater purification is the biochemical activity of microbes, well described in the literature. It has been found that graphene-based materials may have bactericidal activity, with a strong cytotoxic effect on both Gram-positive and Gram-negative bacteria as well as fungi [28]. Graphene oxide consists of stacked sheets with rich oxygen-functional groups including hydroxyl, epoxide, carbonyl, and carboxyl groups. Therefore, cell membranes of the bacteria can be damaged when they are in physical contact with the oxygenated graphene walls [29]. Such nanoparticles may effectively disrupt key metabolic pathways within microorganism cells [30].

**Figure 5:**
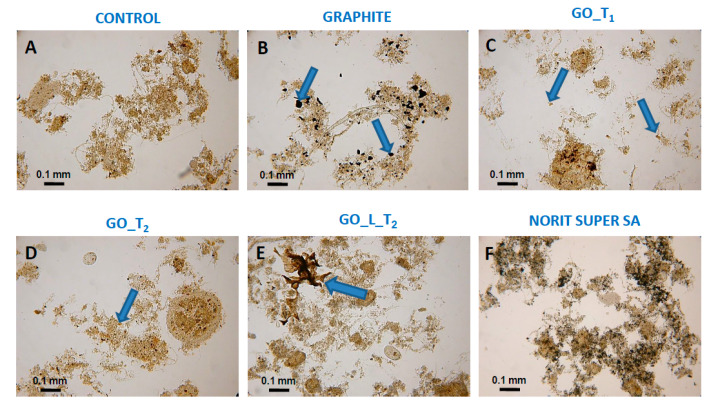
Optical microscope view of activated sludge for samples containing selected carbon materials (dose 0.5 g/L) after reaction (time = 24 h). The arrows show the particles of the dosed carbon nanoparticles. (**A**)—Activated sludge (AC); (**B**)—AC with graphite; (**C**)—AC with graphene oxide GO_T_1_; (**D**)—AC with graphene oxide GO_T_2_; (**E**)—AC with graphene oxide after lyophilization; (**F**)—AC with activated carbon Norit SA Super.

Figure 5 combines representative photographs of the reaction mixture after 24 h of wastewater treatment jar test. Optical microscope views have revealed the incorporation of carbon materials into the structure of activated sludge flocs in tested samples. As can be seen, the carbon particles may easily penetrate the activated sludge flocs, especially GO in suspension form (GO_T_1_ and GO_T_2_). A fraction of the graphite particles also settled well within the floc structures, whilst activated carbon (Norit SA Super) particles were completely included in the activated sludge structures. Interestingly lyophilized graphene oxide GO_L was retained as large solid particles. This was an unexpected effect because good wetting of the GO_L sponge and its spontaneous spread in water were observed previously [13]. Because this material was not sonicated before mixing with the reaction mixture, incomplete dispersion was possible.

Carbon material additives and experiment duration influenced the property of activated sludge (Table 4). A close look for univariate parameters (variables) shows a deterioration of the sedimentation properties of activated sludge in GO containing samples (both liquid forms, GO_T_1_ and GO_T_2_). This effect was measured by SVI and SSV variables. Moreover, we detected a decrease in the value of oxygen uptake by bacteria (DOUR values). This may indicate the negative impact of GO_T_1_ and GO_T_2_ additives on the morphology and antibacterial activity of graphene oxide based on oxidative stress, as was observed by other authors [28,30]. Capillary suction time (CST) values revealed improvement of activated sludge properties for GO_T_2,_ GO_L_T_2_ and Norit Super SA additives (lowest CST values were recorded).

Data in Table 5 consist of the measured values of the physicochemical parameters of wastewater recorded using the jar test. As can be seen, a similar trend in the changes of the analyzed parameter values are visible in comparison with data presented in Table 3 (experimental protocol without activated sludge). Predominantly, the pH and conductivity values decreased in graphene oxide GO_T_1_ and GO_T_2_ modified samples. A similar effect can be observed for the concentration of total nitrogen, although the differences between samples were smaller than recorded previously in the test without activated sludge.

In the presence of activated sludge both orthophosphates and ammonia nitrogen concentrations decreased significantly due to the biological removal of these compounds. All GO additives strongly influenced inorganic carbon contents (IC). The values of this parameter decreased in comparison to control and remaining carbon materials. A similar effect was recorded in the previous jar test (data presented in Table 3). Results of TOC analysis revealed decrease of total organic carbon values in the case of Norit Super SA additive.

### 3.3. Multivariate Data Mining

In principle, the univariate approach for data mining of complex systems is strongly limited [31]. This problem is mainly visible if biological systems are studied. The most commonly used multivariate approach enabling us to determine the number of important factors (variables) affecting the object’s (sample’s) behavior, as well as relationships between the objects, is principal components analysis (PCA). In this study we used PCA to determine possible latent information that was difficult to reveal from the initial univariate raw data set described above in parts 3.2.1. and 3.2.2. Principal component analysis may capture the essential data patterns from the raw data set and object classification.

**Figure 6:**
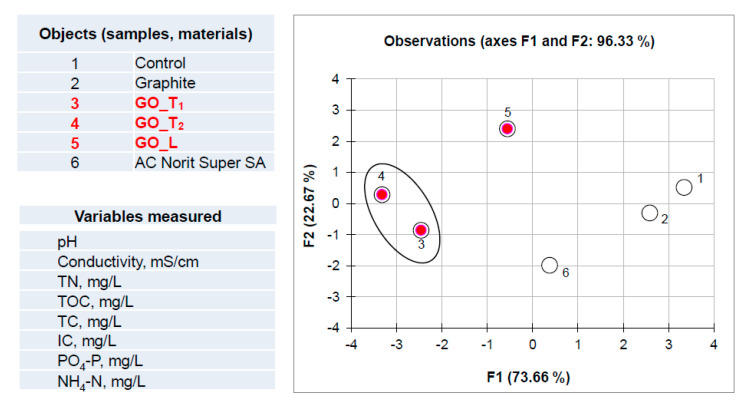
Results of principal component analysis (PCA) (F1/F2 factor scores plot) using matrix consisting of wastewater parameters measured (variables) after reaction (time 24 h) with selected carbon materials (objects).

The starting matrix for PCA study was a data set consisting of 48 measurements (raw data included in Table 3 concerning results of the experiment based on carbon materials and wastewater; part 3.2.1). The computation matrix was organized as six objects (control mixture and samples consisting of given carbon materials) x eight variables (physicochemical parameter values). The number of PC that may characterize this data set was estimated taking into account eigenvalues greater than 1 (Kaiser criterion). For this case, the first two factors (F1 and F2) were found to describe more than 96% of the total variability. Analysis of calculated factor loadings data (non-presented) has revealed that object clustering presented in Figure 6 is mainly driven by variables No 2, 3, 5 and 6, which affect the object spread along the most important F1 axis (73.66% of total variability). As can be seen from the graph in Figure 6, the effect of graphene oxide in liquid forms (GO_T1; label 3 and GO_T2; label 4) on wastewater parameters can be different than the remaining carbon particles, since they are clustering away from the others objects, considering the F1 factor. Interestingly, graphite particles (label 2) do not affect the wastewater parameters; this object is close to the control sample (label 1).

**Figure 7:**
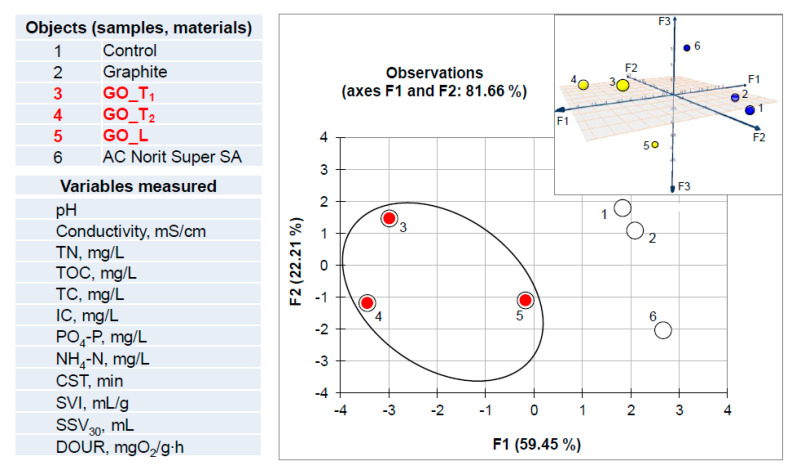
Projection of the objects set in 2D and 3D factor score spaces concerning PCA analysis of data matrix, related to the experiment involving wastewater and activated sludge with carbon materials.

**Figure 8:**
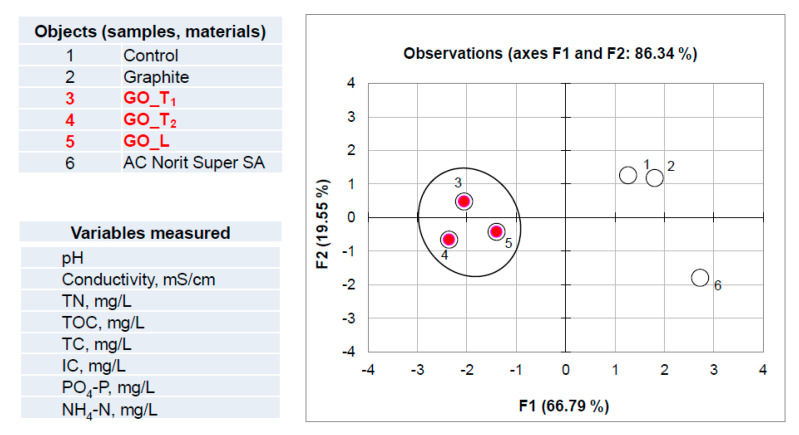
Projection of the objects set in 2D factor score space concerning PCA analysis of data matrix consisting of physicochemical parameters of wastewater, related to the experiment involving wastewater and activated sludge with carbon materials.

The graph in Figure 7 corresponds to PCA analysis of data from the experiment involving addition of activated sludge. The presence of biomass creates a more complicated system than described above. The raw data set used for multivariate computation was derived from Table 4 and Table 5. In this case the raw data set included the values of activated sludge parameters combined with recorded values of physicochemical wastewater parameters. Initial matrix consisted of 72 measurements (12 variables x 6 objects). PCA analysis revealed that for the above matrix the factors F1 and F2 describe more than 81% of the total variability. According to the Kaiser criterion the F3 seems also to be important (cumulative variability for F1/F2/F3 is equal to 96.2%; F3 counts for 14.56%). Based on this analysis and object clustering in both 2D and 3D spaces visible in Figure 7, it can be concluded that all GO materials investigated may affect the experimental mixture (wastewater and activated sludge). This is confirmed by data re-calculation consisting of the values of wastewater physicochemical parameters, exclusively (Figure 8). As can be seen the clustering of samples labeled as 3, 4 and 5 revealed the same effect for all GO materials investigated.

## 4. Conclusions

Presented studies have revealed that investigated graphene oxide materials are non-stable during storage within a 36 week time period from synthesis. It was demonstrated that all GO materials are active and may affect pollutants presented in wastewater via direct interaction or by affecting biological processes of activated sludge.

Univariate measurements clearly indicated that GO in suspension form may act in a different way in comparison to the remaining carbon particles (graphite, activated carbon). Particularly, a decrease in pH, conductivity, TOC, IC, and ammonia as well as total nitrogen was recorded. In addition, GO in suspension form demonstrated the negative effect on measured parameters of activated sludge, namely SVI and DOUR. Different behavior of lyophilized GO may result from incomplete dispersion in the reaction mixture.

Multivariate analysis of data obtained from both experimental setups (wastewater and wastewater with activated sludge jar tests) may suggest that all GO materials investigated may affect physicochemical and biological processes of wastewater treatment.

## Figures and Tables

**Table 1:** Energy dispersive spectroscopy (EDS) elemental analysis of carbon materials investigated.

Elements	Graphite		Norit SA Super		GO_AD		GO_L	
	Wt. %	At. %	Wt. %	At. %	Wt. %	At. %	Wt. %	At. %
C	100	100	90.28	94.38	47.33	55.30	54.01	61.51
O			5.01	3.93	49.27	43.21	44.05	37.66
S					3.40	1.49	1.94	0.83
Mg			0.91	0.47				
Al			0.47	0.22				
Si			0.54	0.24				
Ca			1.51	0.47				
Fe			1.28	0.29				

GO_AD—graphene oxide after air drying; GO_L—graphene oxide after lyophilization; Wt—Weight; At—Atomic.

**Table 2:** Intensity ratio of D/G (I_D/G_) and 2D/G (I_2D/G_) bands calculated from Raman spectra (Figure 4) of the carbon-based materials.

Material	I_D/G_	I_2D/G_
Graphite	0.10	0.35
Norit SA Super	1.04	0.17
GO_C	0.26	0.30
GO_T_1_	0.88	0.13
GO_T_2_	0.83	0.19
GO_AD	0.80	0.12
GO_L_T_2_	0.78	0.18

**Table 3:** Results of wastewater physicochemical analysis after 24 h reaction with selected carbon materials (dose 0.5 g/L).

Material	Parameter							
	pH	Conductivity	TN	TOC	TC	IC	Orthophosphates	Ammonia Nitrogen
	-	mS/cm	mg/L	mg/L	mg/L	mg/L	mg/L	mg/L
Control sewage sample without carbon additives	8.45	1.546	20.75	74.46	165.40	90.90	12.0	36.8
Graphite	8.37	1.553	19.87	67.25	157.30	90.02	11.0	33.8
GO_T_1_	8.23	1.466	16.96	34.46	98.85	64.39	13.0	28.0
GO_ T_2_	8.13	1.468	17.02	31.69	87.29	55.59	15.0	29.2
GO_L_ T_2_	8.14	1.503	19.78	63.69	125.80	62.15	16.0	34.2
Norit Super SA	8.42	1.533	18.70	33.96	123.00	89.02	12.0	28.2

TN—Total Nitrogen; TOC—Total Organic Carbon; TC—Total Carbon; IC—Inorganic Carbon.

**Table 4:** Selected parameter values of activated sludge after 24 h reaction with carbon materials (dose 0.5 g/L).

Material	Activated Sludge Parameters *			
	CST	SVI	SSV_30_	DOUR
	min	mL/g	ml/L	mgO_2_/g∙h
Control activated sludge sample without carbon additives	10.15	100	380	27.6
Graphite	10.27	84	320	21.8
GO_T_1_	12.12	210	800	20.5
GO_T_2_	8.88	224	850	14.9
GO_L_T_2_	7.57	92	350	22.3
Norit Super SA	7.05	97	370	20.5

* SSV_30_—settled sludge volume after 30 min; DOUR—dissolved oxygen uptake rate; SVI—sludge volumetric index; CST—capillary suction time.

**Table 5:** Results of wastewater physicochemical analysis after 24 h reaction with activated sludge and selected carbon materials (dose 0.5 g/L).

Material	Parameter							
	pH	Conductivity	TN	TOC	TC	IC	Orthophosphates	Ammonia Nitrogen
	-	mS/cm	mg/L	mg/L	mg/L	mg/L	mg/L	mg/L
Control sewage + activated sludge sample without carbon additives	7.33	1.348	15.57	12.20	29.26	17.06	0.0	0.0
Graphite	7.38	1.364	15.85	12.30	29.65	17.35	0.0	0.0
GO_T_1_	7.08	1.308	14.48	11.11	15.97	4.86	0.0	0.0
GO_T_2_	6.86	1.342	14.34	10.33	13.09	2.76	0.0	0.0
GO_L_T_2_	6.72	1.373	15.21	11.70	15.49	3.79	0.0	0.0
Norit Super SA	7.42	1.391	15.46	4.44	27.59	23.15	0.0	0.0
